# Supplementary material for: The Interaction between CLSPN Gene Polymorphisms and Alcohol Consumption Contributes to Oral Cancer Progression
Source: Int J Mol Sci. 2024 Jan 16;25(2):1098. doi: 10.3390/ijms25021098 (PMC10816373; doi:10.3390/ijms25021098)
Supplement: Supplementary file 1 [file ijms-25-01098-s001.zip › ijms-2726901-supplementary.pdf]

Supplementary Table S1

The distribution of CLSPN SNP genotype frequencies among alcohol drinkers in control and OSCC groups.

Variable	Control (N=8)	Patients (N=116)	AOR [a] (95% CI)	*p* Value
**rs12058760**				
CC	7 (87.5%)	101 (87.1%)	1.000 (reference)	
CG	1 (12.5%)	15 (12.9%)	0.790 (0.068–9.201)	0.850
GG	-	-	-	-
CG+GG	1 (12.5%)	15 (12.9%)	0.790 (0.068–9.201)	0.850
**rs16822339**				
AA	5 (62.5%)	63 (54.3%)	1.000 (reference)	
AC	3 (37.5%)	43 (37.1%)	0.800 (0.142–4.492)	0.800
CC	0 (0.0%)	10 (8.6%)	-	-
AC + CC	3 (37.5%)	53 (45.7%)	1.006 (0.187–5.409)	0.995
**rs535638**				
CC	6 (75.0%)	70 (60.3%)	1.000 (reference)	
CT	2 (25.0%)	44 (37.9%)	1.543 (0.250–9.531)	0.640
TT	0 (0.0%)	2 (1.7%)	-	-
CT + TT	2 (25.0%)	46 (39.7%)	1.557 (0.252–9.615)	0.633
**rs7520495**				
CC	3 (37.5%)	12 (10.3%)	1.000 (reference)	
CG	5 (62.5%)	87 (75.0%)	3.633 (0.562–23.485)	0.176
GG	0 (0.0%)	17 (14.7%)	-	-
CG + GG	5 (62.5%)	104 (89.7%)	4.315 (0.674–27.643)	0.123

N: number. [a] The adjusted odds ratio (AOR) with their 95% confidence intervals were estimated by multiple logistic regression models after controlling for betel nut chewing and tobacco consumption. * p value < 0.05 as statistically significant.

Supplementary Table S2

The distribution of CLSPN SNP genotype frequencies among non-alcohol drinkers in control and OSCC groups.

Variable	Control (N=296)	Patients (N=286)	AOR [a] (95% CI)	*p* Value
**rs12058760**				
CC	261 (88.2%)	259 (90.6%)	1.000 (reference)	
CG	34 (11.5%)	25 (8.7%)	0.791 (0.369–1.696)	0.547
GG	1 (0.3%)	2 (0.7%)	4.124 (0.295–57.74)	0.293
CG+GG	35 (11.8%)	27 (9.4%)	0.886 (0.428–1.834)	0.744
**rs16822339**				
AA	177 (59.8%)	153 (53.5%)	1.000 (reference)	
AC	100 (33.8%)	120 (42.0%)	1.133 (0.704–1.824)	0.606
CC	19 (6.4%)	13 (4.5%)	1.384 (0.557–3.438)	0.484
AC + CC	119 (40.2%)	133 (46.5%)	1.169 (0.744–1.837)	0.499
**rs535638**				
CC	203 (68.6%)	177 (61.9%)	1.000 (reference)	
CT	83 (28.0%)	101 (35.3%)	1.309 (0.807–2.125)	0.275
TT	10 (3.4%)	8 (2.8%)	1.016 (0.271–3.810)	0.982
CT + TT	93 (31.4%)	109 (38.1%)	1.280 (0.800–2.047)	0.303
**rs7520495**				
CC	118 (39.9%)	102 (35.7%)	1.000 (reference)	
CG	138 (46.6%)	140 (49.0%)	1.244 (0.762–2.031)	0.382
GG	40 (13.5%)	44 (15.4%)	1.381 (0.688–2.774)	0.364
CG + GG	178 (60.1%)	184 (64.3%)	1.274 (0.799–2.030)	0.309

N: number. [a] The adjusted odds ratio (AOR) with their 95% confidence intervals were estimated by multiple logistic regression models after controlling for betel nut chewing and tobacco consumption. * p value < 0.05 as statistically significant.

Supplementary Table S3

The distribution of CLSPN SNP genotype frequencies among betel nut eaters in control and OSCC groups.

Variable	Control (N=11)	Patients (N=258)	AOR [a] (95% CI)	*p* Value
**rs12058760**				
CC	9 (81.8%)	232 (89.9%)	1.000 (reference)	
CG	2 (18.2%)	26 (10.1%)	0.405 (0.080–2.058)	0.276
GG	-	-	-	-
CG+GG	2 (18.2%)	26 (10.1%)	0.405 (0.080–2.058)	0.276
**rs16822339**				
AA	5 (45.5%)	134 (51.9%)	1.000 (reference)	
AC	6 (54.5%)	111 (43.0%)	0.700 (0.206–2.375)	0.568
CC	0 (0.0%)	13 (5.0%)	-	-
AC + CC	6 (54.5%)	124 (48.1%)	0.756 (0.223–2.563)	0.654
**rs535638**				
CC	7 (63.6%)	154 (59.7%)	1.000 (reference)	
CT	4 (36.4%)	97 (37.6%)	1.079 (0.305–3.819)	0.906
TT	0 (0.0%)	7 (2.7%)	-	-
CT + TT	4 (36.4%)	104 (40.3%)	1.163 (0.329–4.109)	0.815
**rs7520495**				
CC	4 (36.4%)	71 (27.5%)	1.000 (reference)	
CG	5 (45.5%)	148 (57.4%)	1.144 (0.289–4.526)	0.848
GG	2 (18.2%)	39 (15.1%)	0.837 (0.142–4.923)	0.844
CG + GG	7 (63.6%)	187 (72.5%)	1.054 (0.292–3.809)	0.936

N: number. [a] The adjusted odds ratio (AOR) with their 95% confidence intervals were estimated by multiple logistic regression models after controlling for alcohol and tobacco consumption. * p value < 0.05 as statistically significant.

Supplementary Table S4

The distribution of CLSPN SNP genotype frequencies among non-betel nut eaters in control and OSCC groups.

Variable	Control (N=293)	Patients (N=144)	AOR [a] (95% CI)	*p* Value
**rs12058760**				
CC	259 (88.4%)	128 (88.9%)	1.000 (reference)	
CG	33 (11.3%)	14 (9.7%)	0.941 (0.439–2.016)	0.875
GG	1 (0.3%)	2 (1.4%)	4.232 (0.297–60.29)	0.287
CG+GG	34 (11.6%)	16 (11.1%)	1.040 (0.502–2.154)	0.916
**rs16822339**				
AA	177 (60.4%)	82 (56.9%)	1.000 (reference)	
AC	97 (33.1%)	52 (36.1%)	1.228 (0.746–2.021)	0.420
CC	19 (6.5%)	10 (6.9%)	1.443 (0.574–3.628)	0.435
AC + CC	116 (39.6%)	62 (43.1%)	1.261 (0.786–2.022)	0.337
**rs535638**				
CC	202 (68.9%)	93 (64.6%)	1.000 (reference)	
CT	81 (27.6%)	48 (33.3%)	1.360 (0.820–2.255)	0.234
TT	10 (3.4%)	3 (2.1%)	0.941 (0.226–3.915)	0.933
CT + TT	91 (31.1%)	51 (35.4%)	1.317 (0.806–2.152)	0.272
**rs7520495**				
CC	117 (39.9%)	43 (29.9%)	1.000 (reference)	
CG	138 (47.1%)	79 (54.9%)	1.344 (0.797–2.268)	0.267
GG	38 (13.0%)	22 (15.3%)	1.783 (0.872–3.647)	0.113
CG + GG	176 (60.1%)	101 (70.1%)	1.435 (0.873–2.358)	0.154

N: number. [a] The adjusted odds ratio (AOR) with their 95% confidence intervals were estimated by multiple logistic regression models after controlling for alcohol and tobacco consumption. * p value < 0.05 as statistically significant.

Supplementary Table S5

The distribution of CLSPN SNP genotype frequencies among smokers in control and OSCC groups.

Variable	Control (N=23)	Patients (N=312)	AOR [a] (95% CI)	*p* Value
**rs12058760**				
CC	21 (91.3%)	279 (89.4%)	1.000 (reference)	
CG	2 (8.7%)	32 (10.3%)	1.082 (0.233–5.021)	0.920
GG	0 (0.0%)	1 (0.3%)	-	-
CG+GG	2 (8.7%)	33 (10.6%)	1.190 (0.257–5.503)	0.824
**rs16822339**				
AA	13 (56.5%)	167 (53.5%)	1.000 (reference)	
AC	10 (43.5%)	129 (41.3%)	0.928 (0.383–2.249)	0.868
CC	0 (0.0%)	16 (5.1%)	-	-
AC + CC	10 (43.5%)	145 (46.5%)	1.044 (0.433–2.516)	0.924
**rs535638**				
CC	15 (65.2%)	193 (61.9%)	1.000 (reference)	
CT	8 (34.8%)	111 (35.6%)	0.981 (0.393–2.448)	0.967
TT	0 (0.0%)	8 (2.6%)	-	-
CT + TT	8 (34.8%)	119 (38.1%)	1.048 (0.421–2.610)	0.920
**rs7520495**				
CC	9 (39.1%)	85 (27.2%)	1.000 (reference)	
CG	11 (47.8%)	180 (57.7%)	1.527 (0.583–3.996)	0.389
GG	3 (13.0%)	47 (15.1%)	1.349 (0.335–5.429)	0.673
CG + GG	14 (60.9%)	227 (72.8%)	1.486 (0.596–3.706)	0.395

N: number. [a] The adjusted odds ratio (AOR) with their 95% confidence intervals were estimated by multiple logistic regression models after controlling for betel nut chewing and alcohol consumption. * p value < 0.05 as statistically significant.

Supplementary Table S6

The distribution of CLSPN SNP genotype frequencies among non-smokers in control and OSCC groups.

Variable	Control (N=281)	Patients (N=90)	AOR [a] (95% CI)	*p* Value
**rs12058760**				
CC	247 (87.9%)	81 (90.0%)	1.000 (reference)	
CG	33 (11.7%)	8 (8.9%)	0.760 (0.320–1.809)	0.536
GG	1 (0.4%)	1 (1.1%)	3.820 (0.236–61.90)	0.346
CG+GG	34 (12.1%)	9 (10.0%)	0.848 (0.372–1.930)	0.694
**rs16822339**				
AA	169 (60.1%)	49 (54.4%)	1.000 (reference)	
AC	93 (33.1%)	34 (37.8%)	1.204 (0.701–2.070)	0.502
CC	19 (6.8%)	7 (7.8%)	1.343 (0.505–3.573)	0.554
AC + CC	112 (39.9%)	41 (45.6%)	1.227 (0.735–2.047)	0.434
**rs535638**				
CC	194 (69.0%)	54 (60.0%)	1.000 (reference)	
CT	77 (27.4%)	34 (37.8%)	1.463 (0.851–2.516)	0.169
TT	10 (3.6%)	2 (2.2%)	0.873 (0.185–4.124)	0.864
CT + TT	87 (31.0%)	36 (40.0%)	1.397 (0.824–2.369)	0.214
**rs7520495**				
CC	112 (39.9%)	29 (32.2%)	1.000 (reference)	
CG	132 (47.0%)	47 (52.2%)	1.262 (0.715–2.229)	0.422
GG	37 (13.2%)	14 (15.6%)	1.721 (0.805–3.677)	0.161
CG + GG	169 (60.1%)	61 (67.8%)	1.361 (0.795–2.329)	0.262

N: number. [a] The adjusted odds ratio (AOR) with their 95% confidence intervals were estimated by multiple logistic regression models after controlling for betel nut chewing and alcohol consumption. * p value < 0.05 as statistically significant.
